# Factors associated with postpartum fatigue: an exploration of the moderating role of resilience

**DOI:** 10.3389/fpubh.2024.1394380

**Published:** 2024-06-14

**Authors:** Baian A. Baattaiah, Mutasim D. Alharbi, Monira I. Aldhahi, Fayaz Khan

**Affiliations:** ^1^Department of Physical Therapy, Faculty of Medical Rehabilitation Sciences, King Abdulaziz University, Jeddah, Saudi Arabia; ^2^Department of Rehabilitation Sciences, College of Health and Rehabilitation Sciences, Princess Nourah bint Abdulrahman University, Riyadh, Saudi Arabia

**Keywords:** postpartum fatigue, resilience, depression symptoms, sleep quality, maternal health

## Abstract

**Background:**

Postpartum fatigue (PPF) can impair the physical and mental well-being of women. The aims of this study were to assess the associations between fatigue and maternal health-related variables, specifically, sleep quality, depression symptoms, and resilience, and to explore the moderating role of resilience in the relationships between sleep quality, depression symptoms, and fatigue.

**Methods:**

This cross-sectional study used data collected from mothers during the postpartum period via an online platform. PPF was assessed using the Fatigue Severity Scale, whereas sleep quality and depression symptoms were assessed using the Pittsburgh Sleep Quality Index and Edinburgh Postnatal Depression Scale, respectively. The Brief Resilience Scale was used to assess resilience. Simple and multiple binary logistic regression analyses were performed to examine the association of each independent variable with PPF and to determine the most significant predictors of PFF. The data were analyzed using SPSS, and structural equation modeling was performed using AMOS 23. A moderation analysis was performed to explore the moderating role of resilience using the Hayes PROCESS macro.

**Results:**

A total of 1,443 postpartum mothers were included in the analysis. The simple binary logistic regression analysis showed that having chronic disease (odds: 1.52; *p* = 0.02), mother’s age (odds: 0.97; *p* = 0.03), mother’s body mass index (BMI; odds: 1.03; *p* = 0.01), depression symptoms (odds: 1.09; *p* ≤ 0.0001), sleep quality (odds: 1.17; *p* ≤ 0.0001), and resilience (odds: 0.42; p ≤ 0.0001) all contributed to fatigue during postpartum. Multivariate logistic regression showed that the mother’s BMI, sleep quality, depression symptoms, and resilience were significant predictors of PPF. Moderation analyses showed that resilience was not a significant moderator between the main effects of sleep quality and fatigue (interaction effect: *β* = 0.01, *p* = 0.31, 95% CI: −0.01 to 0.04) or between the main effects of depression symptoms and fatigue during postpartum (interaction effect: *β* = 0.01, *p* = 0.82, 95% CI: −0.01 to 0.02).

**Conclusion:**

Given the deleterious effects of PPF on maternal health outcomes, factors associated with PPF should be assessed regularly. In addition to mothers’ BMI, sleep quality, and depression symptoms, resilience could also be a crucial factor in predicting fatigue severity during this critical time for mothers even though it was not a significant moderator among this sample.

## 1 Introduction

The postpartum period necessitates continuous and thorough care, as mothers are at risk of developing fatigue, depression, and other health-related issues. In general, fatigue hampers quality of life and may affect individuals across the life spectrum (1–3). Notably, new mothers are particularly prone to postpartum fatigue (PPF), which is characterized by a prolonged decrease in physical and mental activity, energy loss, and impaired concentration following childbirth (4–6). PPF is often perceived as a consequence of the physical adaptations and demands of motherhood (7, 8). The proportion of new mothers reporting fatigue was 37–64% at 5–6 weeks, 25–67% at 12–24 weeks, and 18–66% at 1–2 years after childbirth (9, 10). It has been reported that PPF increases anxiety (11), diminishes the mother’s sense of self-esteem in parenting (12), and causes depression (13), which can eventually affect mother–infant bonding and/or overall health.

Literature related to the postpartum period has investigated potential risk factors for initial and persistent fatigue among mothers (9, 14–16). Research has explored depression, sleep, anxiety, maternal age, socioeconomic status, educational status, employment status, and marital status and breastfeeding (i.e., maternal health-related factors) in relation to PFF. A previous study investigating the factors associated with fatigue occurring within 3 months postpartum has highlighted that younger age and greater socioeconomic disadvantage, lower self-efficacy, and poorer sleep quality contribute to earlier fatigue, whereas the stability of fatigue over time is attributed to older maternal age and poor sleep quality (14). Another study has also identified significant associations between demographic and socioeconomic factors, the health statuses of both mothers and children, and the levels of fatigue experienced by postpartum mothers. Data has demonstrated that maternal age during the postpartum period plays a predictive role in PPF (17). Specifically, younger mothers have been found to be at a significantly lower risk of experiencing PPF than older mothers (15). These findings underscore the influence of these variables on PPF risk and severity. Additionally, maternal obesity and overweight have been identified as significant risk factors for the development of PPD, which may subsequently contribute to increased fatigue levels during the postpartum period (18). Thus, common chronic health conditions in women have been identified as exacerbating factors owing to their physiological, psychological, and lifestyle impacts (19, 20). This finding suggests that the complex interplay between these conditions may intensify PPF. However, previous studies have not thoroughly examined the interrelationships between demographic characteristics and physical and maternal health-related factors, including sleep quality and psychological health factors, in relation to the severity of PPF.

Sleep disturbance has been independently studied as the main risk factor for fatigue among postpartum women, secondary to the responsibilities of motherhood. Sleep disturbance is a serious problem linked to depression, anxiety disorders, and stress (21–23). On average, women postnatally exhibit increased baseline melatonin release, decreased maximum melatonin release, decreased percentage of increased melatonin release, and different patterns of melatonin release than non-pregnant nulliparous women, which may put them at risk of circadian rhythm disruption (24). It has been shown that disturbances in the endogenous circadian rhythm among healthy women during the postpartum period are associated with high levels of fatigue (25). This trend suggests that there are individual factors and differences in the fatigue levels among women during the postpartum period. These findings highlight sleep as a crucial factor affecting mothers’ health during the postpartum period; thus, this phenomenon requires extensive investigation and direct intervention.

Postpartum depression (PPD) can occur 4 weeks to 12 months after childbirth (26). Previous evidence has suggested that fatigue and depression are interrelated during the 2 years following childbirth (13, 27). While fatigue and depression are bidirectionally related during the postpartum period, they remain distinct constructs (6, 28, 29). Research has confirmed this univariate relationship, and other researchers have postulated that fatigue could predict depression and vice versa (17, 27, 30, 31). Given this close relationship, the findings of a study by Bozoky and Corwin suggest that persistent fatigue can increase women’s risk of PPD. (27) In addition, another study that assessed the relationship between PPF and depressive symptoms and stress showed that PPF and PPD symptoms were significantly correlated in the first 3 weeks, with PPF at 2 weeks after childbirth acting as the best predictor for the development of PPD. (30) Due to variations in study samples and the direction of the relationship between fatigue and depression in previous research, future studies should build on the extensive evidence to verify that depressive symptoms are likely independent predictors of fatigue among mothers during postpartum.

Psychosocial factors may influence women’s sense of well-being during the postpartum period. Resilience is a personal protective factor against adversity in life (32); it reflects an individual’s adaptability and capacity to cope with stressful situations and adversity (33). Resilient individuals are mainly characterized by self-reliance, emotional regulation, and supportive close relationships that may enable them to maintain normal psychological and physical functioning (34, 35). Significant research has been conducted regarding resilience and depression and their independent associations in postpartum women (36–39). These studies illuminate the impact of resilience and suggest that resilience can counteract PPF. However, it remains unclear whether resilience, an emerging and evolving construct in rehabilitation science (40, 41), protects against fatigue during the postpartum period.

As mentioned, multifaceted factors have been shown to induce PPF. However, the connections between these constructs and the role of resilience as a moderator have not been investigated and are not well understood. Comprehensive analysis is essential to elucidate the association between demographic characteristics, physical and psychological health factors, and the severity of PPF among postpartum mothers. Such an analysis should encompass a range of factors, including demographic characteristics (e.g., age, educational status, employment status, and marital status), physical and maternal health-related factors (including BMI, presence of chronic diseases, term status of pregnancy, number of children, age of the child, and sleep quality), and psychological factors (e.g., symptoms of depression and levels of resilience). A multifaceted approach should aim to clarify the interconnections and impacts of these factors on PPF to provide a clearer understanding for further research and intervention development. Moreover, understanding the moderating role of resilience in the relationships between fatigue and sleep quality or fatigue and PPD may positively affect health-related outcomes during the postpartum period. Identifying the indirect influence of resilience on women’s health outcomes, such as levels of fatigue, could inspire interventions that improve the resilience of mothers during the postpartum period, as women might be at a high risk of fatigue, which is considered a clinically prevalent symptom during this stressful period for mothers that may impact their physical activity and social engagement.

Therefore, this study aimed to assess the association between fatigue and mothers’ health-related variables, specifically, sleep quality, depression symptoms, and resilience. This study also aimed to explore the moderating role of resilience on the relationship between predicting factors (sleep quality and depression symptoms) and fatigue. We hypothesized that PPF is multifactorial, i.e., influenced by physical and maternal health-related determinants as well as psychological factors, including symptoms of depression and levels of resilience. Additionally, we hypothesized that resilience may moderate the relationships between fatigue, sleep quality, and depression during the postpartum period. Early screening for fatigue and its associated factors may enable the identification of mothers susceptible to fatigue, thus minimizing the potential occurrence of non-communicable diseases associated with persistent fatigue later in life if they remain neglected (42–44). The findings of this study underscore the importance of recognizing both potential risk factors for fatigue and the probable role of resilience among women experiencing postpartum fatigue. The enhanced understanding of resilience resulting from this study may improve prevention and treatment strategies for PPF in rehabilitation settings.

## 2 Methods and materials

### 2.1 Study design and sample

This study uses a cross-sectional design with data collected via an electronic survey between February and May 2022. This study was part of a research project concerning the health of mothers during postpartum. All questionnaires for the study were attached to the survey and were distributed through various electronic means (email, X, and WhatsApp). The desired study sample was mothers living in Saudi Arabia, aged 18–45 years, and in their postpartum period (up to 12 months after childbirth).

Prior to data collection, we estimated the sample size required for this study using OpenEpi.com (45). The suggested sample size was 385 participants, considering the total adult female population of Saudi Arabia in 2021 (approximately 10,000,000), a confidence level of 95%, a margin of error of 5%, and an anticipated frequency of 50%. The survey was completed using a convenience sample of 1,443 mothers.

### 2.2 Ethical approval and informed consent

This study was carried out in compliance with the guidelines of the Declaration of Helsinki. The Ethics Committee of the Faculty of Medical Rehabilitation Sciences (FMRS), King Abdulaziz University, Jeddah, Saudi Arabia, reviewed and approved the study procedure (FMRS-EC2022-006). The introductory section of the survey briefly described the rights of the participants and the study protocol. In addition, mothers who wished to participate were asked to agree to the informed consent sentences that were also provided in the introductory section. The principal investigator’s contact information was provided so respondents could reach out with any queries.

### 2.3 Study instrument/measures

A detailed explanation of the study instrumentation is provided in our previous work concerning PPD symptoms (39). Mothers who agreed to participate were asked to complete a survey that included the Fatigue Severity Scale (FSS), Pittsburgh Sleep Quality Index (PSQI), Edinburgh Postnatal Depression Scale (EPDS), and Brief Resilience Scale (BRS) in addition to several sociodemographic and health-related questions. The total time to complete the entire survey was 5–7 min.

#### 2.3.1 Socio-demographic and health- and childbirth-related questions

The sociodemographic questions concerned the participant’s age, weight, height, educational level, marital status, and employment status. Health- and childbirth-related questions concerned the participant’s history of chronic diseases (presence or absence), smoking status, youngest child’s age (their most recent child), mode of delivery for the youngest child, term of pregnancy of the youngest child, number of children excluding the youngest child, and if they were receiving assistance at home during the postpartum period (a house keeper, babysitter, etc.).

#### 2.3.2 Fatigue severity scale

The FSS was established by Krupp et al. (46) to measure fatigue, which is defined as a sense of physical tiredness and lack of energy, distinct from sadness or weakness in people with chronic conditions (47). The FSS is a nine-item scale, with answers rated on a seven-point Likert scale ranging from 1 (strongly disagree) to 7 (strongly agree). The fatigue cutoff score was ≥4 using the mean scores of the items (48–50). The Arabic-translated version of the scale is valid for use by Arabic-speaking populations, as it demonstrates a good score of internal consistency (*α* = 0.84) (51).

#### 2.3.3 Pittsburgh sleep quality index

The PSQI was established by Buysse et al. (52). The scale consists of 19 items intended to measure seven characteristics of sleep during the prior month: sleep quality, sleep onset latency, sleep duration, sleep efficiency, sleep disturbances, sleeping medication use, and daytime dysfunction. Each sleep characteristic is scored from 0 (no difficulty) to 3 (severe difficulty). The total score can range from 0 to 21 points, with higher scores indicating worse sleep quality. A cutoff point of ≥5 indicates poor sleep quality (53, 54). The Arabic-translated version of the scale is valid for use by Arabic-speaking populations, as it has demonstrated adequate validity and acceptable reliability (*α* = 0.65) (55).

#### 2.3.4 Edinburgh postnatal depression scale

The EPDS was established by Cox et al. (56) to identify PPD disorders, which are defined as distressing disorders that last longer than the “blues” but are less severe than postpartum psychosis. The EPDS is considered a screening tool for depression symptoms in both the pre- and postnatal periods (57). The scale consists of 10 items, each of which is scored on a 4-point scale ranging from 0 to 3 based on the severity of symptoms. The Arabic-translated version of the scale is valid for use by Arabic-speaking populations, as it has demonstrated a good internal consistency score (α = 0.84). The total score of the scale can range from 0 to 30 points, with a cutoff point of ≥12 indicating a good score of specificity for PPD symptoms among the Arabic-speaking population (58).

#### 2.3.5 Brief resilience scale

The BRS was established by Smith et al. (59) to assess resilience. It consists of six items, with answers scored from 1 (strongly disagree) to 5 (strongly agree). To calculate the mean, items 2, 4, and 6 of the scale should first be reverse coded and then the mean of all item scores should be taken. The total score of the scale can range from 6 to 30 points, with poor resilience indicated by a score of ≤2.95 and high resilience indicated by a score of ≥3.99 for general adults. Specifically for females, poor resilience is indicated by a score ≤ 2.87, and high resilience is indicated by a score ≥ 3.91 (59, 60). The Arabic-translated version of the scale is valid for use by the Arabic-speaking population, as it has demonstrated excellent internal consistency (*α* = 0.98) (61).

### 2.4 Statistical analysis

The data were analyzed using SPSS version 23 (SPSS, Inc., Chicago, IL, United States), and structural equation modeling was performed using AMOS 23. We examined all collected data during the data-cleaning process, and we did not identify any outliers or missing data pertaining to the study instruments. Descriptive data were expressed as frequency (n) and percentages (%) for categorical variables and as mean ± standard deviation (SD) for continuous variables. A simple binary logistic regression was performed to examine the influence of each independent variable on fatigue. Multiple binary logistic regression using the forward logistic regression method was used to find the most influential factors affecting fatigue during the postpartum period. A moderation analysis was performed using the Hayes PROCESS macro to explore the moderating role of resilience on fatigue. The significance level was *p* ≤ 0.05 for all statistical analyses in this study.

## 3 Results

### 3.1 Demographic characteristics

The demographic characteristics of the participants are shown in Table 1. The mean age of the mothers was 28.87 ± 5.46 years with a mean body mass index (BMI) of 25.75 ± 5.10 kg/m^2^. Most participants (60.4%; 872 participants) reported high levels of fatigue that were above the cutoff point. Of the participants, 95.4% were married and three-quarters had at least a bachelor’s degree (75.3%). The findings revealed that 64.4% were unemployed, and the majority (68.7%) had vaginal childbirth. Our results further showed that most (90.1%) participants had full-term pregnancies, while 75.3% had some kind of assistance during the period of postpartum.

**Table 1:** Demographic and baseline characteristics of the study participants (*N* = 1,443).

Variable	Mean ± SD
Mother’s age	28.87 ± 5.46
Mother’s BMI	25.75 ± 5.10
Child’s age (months)	8.16 ± 4.21
*FSS score	4.28 ± 1.51
PSQI score	10.35 ± 3.37
EPDS score	16.05 ± 6.71
BRS score	3.18 ± 0.73
	Frequency *n* (%)
Marital status	
Married	1,377 (95.4)
Not married	66 (4.6)
Education level	
PhD	20 (1.4)
Master’s	119 (8.2)
Bachelor’s	948 (65.7)
Diploma	107 (7.4)
High school or less	241 (16.7)
No certificate	8 (0.6)
Mother’s work	
Government	157 (10.9)
Private	106 (7.3)
Free business	73 (5.1)
Not working	929 (64.4)
Student	178 (12.3)
Smoking	
Yes	72 (5)
No	1,371 (95)
Chronic disease	
Yes	158 (10.9)
No	1,285 (89.1)
Type of childbirth	
Vaginal	992 (68.7)
Cesarean	451 (31.3)
Full-term pregnancy	
No	142 (9.8)
Yes	1,300 (90.1)
Received assistance	
Yes	1,086 (75.3)
No	357 (24.7)

BMI, body mass index; FSS, Fatigue Severity Scale; PSQI, Pittsburgh Sleep Quality Index; EPDS, Edinburgh Postnatal Depression Scale; BRS, Brief Resilience Scale.

### 3.2 Simple binary logistic regression

Table 2 shows the univariate association between the predictive variables and fatigue during the postpartum period. Mothers with chronic disease (odds: 1.52, 95% CI: 1.07 to 2.17, *p* = 0.02), who had a higher BMI (odds: 1.03, 95% CI: 1.01 to 1.10, *p* = 0.01), and were younger (odds: 0.97, 95% CI: 0.96 to 0.99, *p* = 0.03) reported greater fatigue. Mothers with more children were less fatigued (odds: 0.92, 95% CI: 0.84 to 0.99, *p* = 0.04). Depression symptoms were another factor contributing to fatigue, as mothers with symptoms of depression reported greater fatigue (odds: 1.09, 95% CI: 1.08 to 1.12, *p* ≤ 0.0001). Mothers who reported poorer sleep quality also reported greater fatigue (odds: 1.17, 95% CI: 1.13 to 1.21, *p* ≤ 0.0001). Resilience was another factor contributing to fatigue (odds: 0.42, 95% CI: 0.36 to 0.49, *p* ≤ 0.0001), which indicates that mothers who were less resilient were more prone to fatigue.

**Table 2:** Simple binary logistic regression to assess the factors influencing fatigue during postpartum.

Variables		B	95% CI for B		SE B	β	*p* value
			LL	UL			
Mothers age	Constant	1.05			0.29		
	Age	−0.02	0.96	0.99	0.01	0.97	0.03
Mothers BMI	Constant	−0.26			0.28		
	BMI	0.03	1.01	1.10	0.01	1.03	0.01
Marital status	Constant	0.06			0.24		
	Married	0.38	0.89	2.40	0.25	1.46	0.13
	Not married	Reference					
Smoking	Constant	0.42			0.25		
	Yes	0.03	0.63	1.67	0.05	1.03	0.90
	No	Reference					
Chronic disease	Constant	0.38			0.06		
	Yes	0.42	1.07	2.17	0.18	1.52	0.02*
	No	Reference					
Type of childbirth	Constant	0.39			0.06		
	Cesarean	0.10	0.88	1.39	0.12	1.12	0.39
	Vaginal	Reference					
Received assistance	Constant	0.38			0.06		
	No	0.16	0.92	1.50	0.16	1.17	0.20
	Yes	Reference					
Mother’s work	Constant	0.41			0.07		
	Working	0.04	0.84	1.30	0.11	1.04	0.70
	Not working	Reference					
Full-term pregnancy	Constant	0.40			0.17		
	No	0.02	0.72	1.46	0.18	1.03	0.89
	Yes	Reference					
Child’s age (months)	Constant	0.30	0.99	1.04	0.12	1.02	0.25
	Age	0.02			0.01		
Number of children	Constant	0.59			0.10		
	Children	−0.08	0.84	0.99	0.04	0.92	0.04*
PSQI score	Constant	−1.16	1.13	1.21	0.18	1.17	≤0.0001*
	Sleep quality	0.16			0.02		
EPDS score	Constant	−1.06	1.08	1.12	0.15	1.09	≤0.0001*
	Depression	0.09			0.01		
BRS score	Constant	3.20	0.36	0.49	0.28	0.42	≤0.0001*
	Resilience	−0.86			0.08		

*p ≤ 0.05 is significant. SE, Standard Error; B, unstandardized beta “regression coefficient”; β, standardized beta. BMI, Body Mass Index; PSQI, Pittsburgh Sleep Quality Index; EPDS, Edinburgh Postnatal Depression Scale; BRS, Brief Resilience Scale.

### 3.3 Multivariate logistic regression model

Table 3 shows the results of the multivariate logistic regression analysis to assess the most influential factors associated with fatigue during the postpartum period. In the 4-step model, the mother’s BMI (odds: 1.03, 95% CI: 1.002 to 1.05), sleep quality (odds: 1.08, 95% CI: 1.04 to 1.13), depression symptoms (odds: 1.05, 95% CI: 1.03 to 1.07), and resilience (odds: 0.60, 95% CI: 0.50 to 0.73) were significant predictors of PPF with a model fit of McFadden pseudo-r square value of 0.095; *χ*^2^ = 183.21; *p* ≤ 0.001.

**Table 3:** Multivariable logistic regression to assess the most influential factors associated with fatigue during postpartum.

Variable	B	95% CI for B		SE B	*β*	*P* value
		LL	UL			
Step 1						
Constant	−1.05			0.15		
EPDS score	0.09	1.08	1.12	0.01	1.09	≤0.0001
Step 2						
Constant	1.22			0.41		
EPDS score	0.06	1.05	1.09	0.01	1.07	≤0.0001
BRS score	−0.56	0.47	0.69	0.09	0.57	≤0.0001
Step 3						
Constant	0.43			0.45		
PSQI score	0.08	1.04	1.13	0.02	1.08	≤0.0001
EPDS score	0.05	1.03	1.07	0.01	1.05	≤0.0001
BRS score	−0.50	0.50	0.73	0.09	0.60	≤0.0001
Step 4						
Constant	−0.15			0.53		
Mothers BMI	0.02	1.002	1.05	0.53	1.03	0.03
PSQI score	0.08	1.04	1.13	0.02	1.08	≤0.0001
BRS score	−0.51	0.49	0.72	0.09	0.60	≤0.0001
EPDS score	0.05	1.03	1.07	0.01	1.05	≤0.0001

*p* ≤ 0.05 is significant. SE, Standard Error; B, unstandardized beta “regression coefficient”; β: standardized beta. BMI, Body Mass Index; PSQI, Pittsburgh Sleep Quality Index; EPDS, Edinburgh Postnatal Depression Scale; BRS, Brief Resilience Scale.

### 3.4 Structural equation modeling

**Figure 1:**
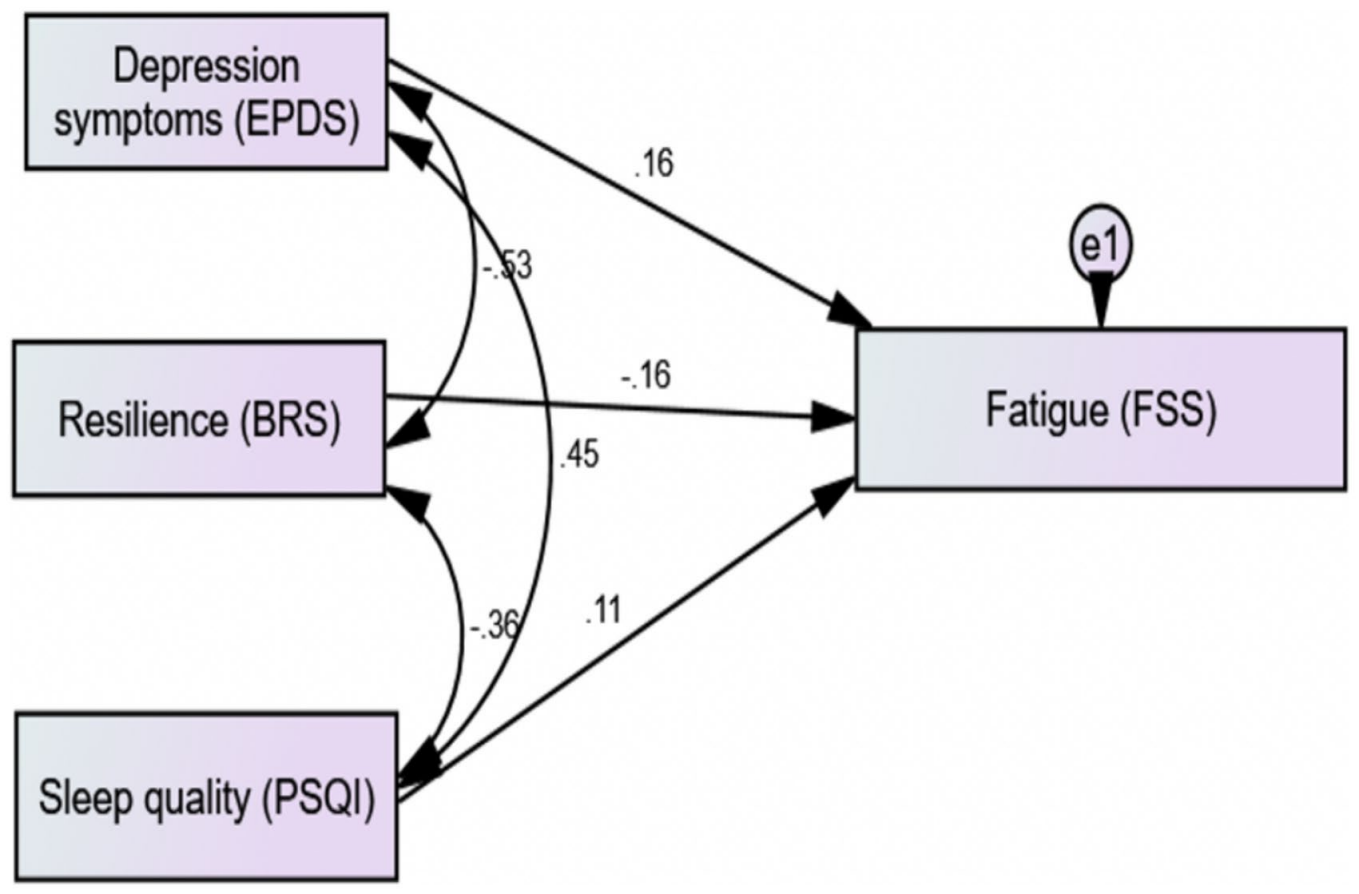
Path diagram of a structural equation model predicting the factors associated with fatigue during postpartum.

Figure 1 illustrates the path diagram of a structural equation model (62) for the predicting factors associated with fatigue during the postpartum period. The standardized regression weights were between fatigue and depression symptoms (estimate: 0.17, *p* ≤ 0.0001), between fatigue and sleep quality (estimate: 0.20, *p* ≤ 0.0001), and between fatigue and resilience (estimate: 0.17, p ≤ 0.0001). The goodness of fit (chi-squared/degree of freedom = 189.84; goodness of fit index = 1.0; comparative fit index = 1.0; root-mean-square error of approximation = 0.36) explains the saturated model.

### 3.5 Moderation analyses

**Figure 2:**
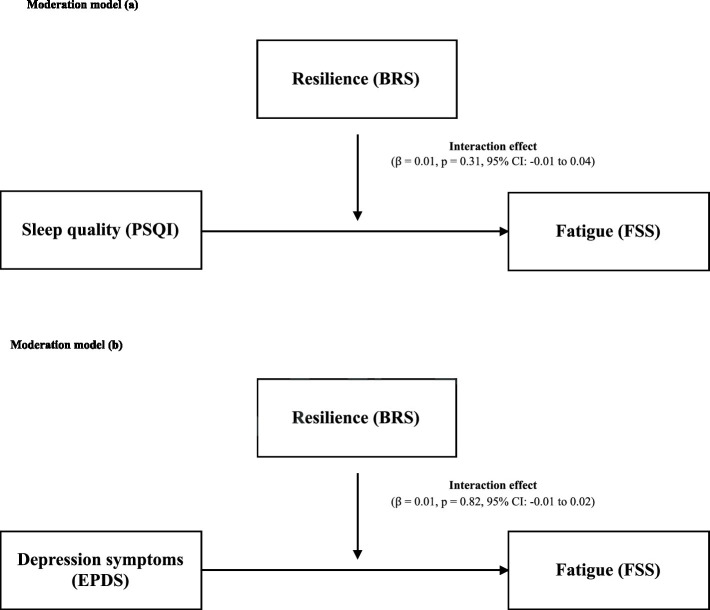
**(A)** Moderation role of resilience in the association between sleep quality and fatigue. **(B)** Moderation role of resilience in the association between depression symptoms and fatigue. BRS, Brief Resilience Scale; PSQI, Pittsburg Sleep Quality Index; FSS, Fatigue Severity Scale; EPDS, Edinburgh Postnatal Depression Scale.

Figure 2 illustrates the moderation analysis. In (a), resilience was included in the model as a moderator between the main effects of sleep quality and fatigue. Our results showed that resilience was not a significant moderator (interaction effect: *β* = 0.01, *p* = 0.31, 95% CI: −0.01 to 0.04). In (b), resilience was included in the model as a moderator between the main effects of depression symptoms and fatigue. Similarly, resilience was not a significant moderator between the main effect of depression symptoms and fatigue (interaction effect: *β* = 0.01, *p* = 0.82, 95% CI: −0.01 to 0.02).

## 4 Discussion

The primary aim of this study was to determine the potential factors associated with PPF, specifically, sleep quality, depression symptoms, and resilience. This study also aimed to explore the moderating role of resilience, as an emerging construct in the context of rehabilitation, on the relationships between fatigue and sleep quality and between fatigue and depression symptoms. Our simple regression analysis revealed that among all factors, a mother’s age, BMI, presence or absence of chronic disease, number of children, sleep quality, depression symptoms, and level of resilience were predictors of the level of fatigue reported by mothers during the postpartum period. In the multiple linear regression analysis, BMI, sleep quality, resilience, and depression symptoms were strong predictors of fatigue. The multivariate direct and/or indirect relationships between the study variables of interest were further confirmed by the structural equation modeling approach. Resilience did not moderate the relationships between fatigue, sleep quality, and depression symptoms. During postpartum, management strategies to mitigate the effects of fatigue on physical performance, mental health, and overall quality of life should be considered. Simple, non-invasive, and cost-effective rehabilitation interventions should be considered to reduce the undesired postpartum-associated symptoms, improve physical activity engagement, and promote the ability and independency of mothers during this critical period. This study is among the first steps in the overarching objective of improving mothers’ health during this critical period because a lack of physical and mental abilities could result in negative mother–infant bonding, diminished overall health, and the eventual development of non-communicable diseases.

The majority (60.4%) of participants reported high levels of fatigue in the FSS. This high percentage is similar to that found in previous studies (9, 10). In contrast, Henderson et al. (15) reported a lower prevalence of PPF among study participants, though PPF was assessed 10 days, 1 month, and 3 months post-partum. This discrepancy between the findings could be attributed to the specific points in the postpartum period in which fatigue was assessed or could be related to the measurement tools used to assess PPF. However, PPF can be multifactorial and is a critical issue among new mothers; PPF can be attributed to medical conditions that occur after delivery, such as anemia, infection, inflammation, thyroid dysfunction, or even hormonal disturbances (63–66). Fatigue during the postpartum period is an etiologically complex phenomenon in which women may feel more uncomfortable and less capable than usual (67, 68). Fatigue is neither less important nor less prevalent than depression among mothers during the postpartum period. Extensive research concerning the underlying pathophysiological causes of PPF should be conducted.

Several studies have found associations between demographics, socioeconomics, the health statuses of both the child and the mother, and fatigue levels among postpartum mothers. A mother’s age during postpartum has been shown to affect predictions of PPF (17). Research has shown that younger mothers were significantly less likely to report PPF (15). This result contrasts with our results in which younger mothers in our sample reported more fatigue symptoms. Our results might imply that younger mothers may not have developed the skills and strategies for dealing with a new child or deescalating or preventing the possible negative consequences of postpartum stress compared to those who are older, who may become more easily accustomed to such a change (69). Another explanation could be that older mothers may feel more financially secure, have more stable relationships, and be better prepared to take care of children (70) than younger mothers, which may play a role in their better well-being. Moreover, obesity and overweight among mothers have been shown to contribute to the risk of developing depression during postpartum (18). Yet, the relationship between PPF and BMI warrants further in-depth investigation. Our results show that a higher BMI increases the likelihood of fatigue. This result is supported by previous work, which has shown that those with higher BMIs experienced greater fatigue than those with lower BMIs during the six-minute walk test (71). In addition, another study has shown that people with lower BMIs may resist depression, fatigue, and anxiety (72). Despite the variations of models used in previous studies, research suggests that higher BMIs may compromise the ventilatory function, as a higher BMI impacts lung compliance and causes cascades of events that may result in activity limitations and fatigue (73–75). An increased BMI may also be negatively associated with cardiorespiratory fitness and lower extremity muscular strength (76), which may lead to activity limitation, exercise intolerance, and disability (77–79). While BMI likely affects fatigue, BMI’s relationship with PPF was not the main focus of this study. Future work should investigate the cellular and physiological mechanisms of BMI in relation to PPF to describe their relationship in detail, as data related to BMI should be interpreted cautiously.

Our results show that chronic diseases were a statistically significant factor in predicting fatigue during the postpartum period. In general, fatigue is associated with several chronic conditions common among women. Fatigue is prevalent among women with diabetes, which could be attributed to several physiological, psychological, and lifestyle factors caused by the disease itself that could predispose individuals to high levels of fatigue (19, 20). In addition, Vitamin D deficiency has been reported to be higher in women than in men (80) and has also been associated with high levels of fatigue (81). In addition, fatigue is a very common side effect of breast cancer, as the disease may affect hormone levels in women’s bodies and eventually lead to fatigue (82). Together, these results indicate that chronic diseases among mothers likely increase levels of fatigue (63, 64, 83), which implies that fatigue could be exacerbated during the postpartum period by the complex interaction between associated factors. However, our study did not ask participants to specify which chronic diseases they might have experienced. Considering that fatigue is significantly detrimental during this period for mothers, and it may have far-reaching negative consequences, further research should elaborate on the most significant chronic conditions that could aggravate the tendency of PPF.

The number of children a mother has was also associated with fatigue in our study. It seems likely that mothers who care for more than one child, in addition to their work, housework, or other responsibilities, would be more tired, as they may experience disrupted sleep and greater burdens and demands (84–86). Our findings might also suggest that those who have experience with previous children might be more accustomed to dealing with new family members (87); however, there is a lack of studies directly focusing on the relationship between PPF and the number of children a mother has. Previous research has shown the relationship between primiparas or multiparas and fatigue at specific times postpartum. The trajectories of the relationships between primiparas with fatigue and multiparas with fatigue were similar in that parity was associated with the course of maternal fatigue during the initial 6 months postpartum. However, primiparas had significantly higher levels of fatigue than multiparas during hospitalization and up to approximately 1 month postpartum. In contrast, multiparas showed significantly higher levels of fatigue than younger primiparas at 6 months postpartum (16). These results indicate that maternal age and experience with having multiple children could be associated with the development of PPF later in the postpartum period. Future studies should emphasize the association between parity and fatigue at specific periods of the postpartum. Such studies may help develop antenatal/postnatal rehabilitation techniques that promote the mental and physical health of mothers.

Similar to previous research, our results show a predictive relationship between sleep quality and mothers’ reported levels of fatigue. Similar results have been extensively addressed in previous research concerning the health of postpartum women, and several interpretations for this phenomenon have been offered. It has been well documented that sleep problems are common during the postpartum period as mothers undergo several physiological, psychological, and social changes while balancing the duties of caring for a newborn (88–90), all of which could negatively impact their ability to sleep and therefore lead to exhaustion and tiredness (91, 92). Fatigue is related to fragmented sleep (83), and a mother may be awoken several times overnight to feed the new baby (93). Furthermore, it has also been shown that mothers’ sleep quality is linked to infants’ sleep patterns, which contributes to PPF (94). Therefore, poor sleep quality significantly influences health outcomes by increasing fatigue levels and decreasing general well-being. Thus, regular sleep quality screening for mothers during the postpartum period is recommended, as such screening is beneficial for establishing appropriate rehabilitation protocols that improve fatigue symptoms and other health outcomes (95, 96). However, there is a lack of studies regarding the prevalence of sleep problems and their associated factors among postpartum mothers who live in Saudi Arabia.

Our results reveal a predictable relationship between symptoms of depression and fatigue, which is consistent with several previous studies (27, 97–100). However, the symptoms of fatigue could overlap with those of depression, though they are somewhat distinct. According to a study by Wilson et al. in 2018 examining the associations between depression and fatigue latent factors using confirmatory factor analysis, depression and fatigue are distinct constructs, but they are still related (101); these results were supported by earlier studies by Giallo et al. (5, 29). That said, it is reasonable to assume that incorporating interventions that target depression symptoms during this critical period for mothers could also improve the symptoms of fatigue; both constructs should be accurately diagnosed via thorough differential evaluations. In addition, focused interventions for fatigue, as distinct from depression, are critical for promoting mothers’ physical functioning and overall health (10, 95, 102).

Our findings provide evidence of a relationship between fatigue and resilience, in which resilience predicts fatigue. Resilience, as an emerging construct in rehabilitation, may play a major role in adaptation to stressors. Previous studies have introduced the relationship between symptoms of depression and resilience (39). However, fatigue commonly occurs in mothers with newborns, and it is not a trivial health issue; it could be a disabling factor that precludes physical or mental activity participation, including activities related to daily living (15). An extensive exploration of the potential psychological factors linked to fatigue during the postpartum period is warranted. There is a lack of research on the relationship between PPF and resilience among mothers living in Saudi Arabia. Previous studies have used various models to reveal this relationship. A study investigating the influence of resilience on fatigue in cancer patients confirmed that resilience is a crucial psychological predictor of fatigue (103). To further confirm the existence of such a relationship, physical and mental fatigue have been shown to be inversely correlated with resilience, and this inverse correlation may be increased by external supports (104). However, such research has been conducted among a sample that might be different in nature and possess different characteristics than our sample. Research has shown that low resilience is associated with worse mental health (105), whereas high resilience is associated with better quality of life among patients experiencing depressive episodes (106). Due to the enormous stress mothers must overcome after giving birth and the health issues that could be resulting from giving birth, resilience could be key to rehabilitation to improve health outcomes, such as fatigue, among those mothers. However, future research is necessary to investigate the specific mechanisms underlying resilience in relation to fatigue.

An increasing amount of research using different models (clinical or non-clinical) has established how resilience may predict or moderate sleep quality (107–111). It has been theorized that there is a bidirectional relationship between low resilience and sleep disturbances (112–114). Resilience, however, appears to mitigate the deleterious impact of perceived stress in the early stage of sleep disruptions (115). In addition, resilience might shield against mental health issues such as anxiety and depression. Resilience is a key component of the deconstruction of the biopsychosocial mechanism of mental illness (116). Our research from 2023 has shown that resilience is the strongest factor that predicts depression symptoms among women during postpartum (39). Wingo et al. explored the moderating role of resilience on the severity of depressive symptoms. The study found that resilience moderates the severity of depressive symptoms in individuals exposed to childhood abuse or other traumas both as a main effect and as an interaction with trauma exposure (117). Despite the differences in the models studied, such results show the potential moderating role of resilience. There seems to be a direct relationship between resilience and sleep issues and depression; however, insufficient research has explored the moderating role of resilience in the relationships between sleep quality and depression symptoms with fatigue among women during postpartum.

A moderating factor can alter the direction of influence and/or strength between dependent and independent variables (118). Unexpectedly, our moderation analysis reveals that resilience is not a strong moderator of the reported association between sleep quality and fatigue. In addition, our results show that resilience was not a strong moderator of the relationship between the risk of PPD and fatigue. This could suggest that the relationships between sleep quality and fatigue and between the risk of PPD and fatigue are strong in our sample. Even though these results are merely exploratory in nature, we believe this research can greatly improve the understanding of the role that could be played by resilience. However, our study is limited by the lack of research regarding resilience’s relation to mothers. Further studies investigating resilience during postpartum are highly encouraged.

Alternatively, factors other than resilience, such as environmental factors, could moderate these relationships. Family support, parity, educational level, employment status, and work hours can potentially affect sleep quality, depression symptoms, and fatigue levels (15, 17, 84, 87, 88, 119, 120). However, it was beyond the scope of this study to investigate the moderating role of sociodemographic factors in these relationships. In addition, it was beyond the scope of this study to investigate the direct relationship between sleep quality and resilience and the moderating role of sleep quality in the relationship between the risk of PPD and fatigue. Further research should explore these points.

### 4.1 Clinical implications

This study is among the initial steps toward the broader goal of enhancing maternal health during the critical postpartum period for mothers living in Saudi Arabia. Maternal fatigue might have multifactorial components, including poor sleep quality, symptoms of depression, and psychological factors, such as low resilience. These factors may lead to systemic inflammation and thus contribute to the pathogenesis of fatigue. Thus, rehabilitation interventions such as structured exercise programs, education, or cognitive behavioral therapy either directly or indirectly targeting fatigue or its associated factors are considered simple, non-invasive, and cost-effective techniques that may improve postpartum symptoms to encourage participation in activities and improve mental and physical health to prevent the possible incidence of non-communicable diseases and improve a mother’s cardiovascular fitness and quality of life (121).

For emphasis, resilience is an emerging concept in the field of rehabilitation; resilience is important because it permits individuals to cope with and adapt to new situations and, therefore, maintain daily life activities. Rehabilitation settings intended to increase resilience would encourage individuals to be more accepting and flexible so they can better cope with stressors and comply with the interventions to benefit from the treatment.

### 4.2 Strengths and limitations

In addition to the well-known factors related to fatigue, this study provides empirical evidence for a new psychological construct—resilience—that could be associated with PPF using a large and random population-based sample. Data used in this study were collected from mothers living in various regions of Saudi Arabia. However, this study has some limitations that must be addressed. Our study could have been limited by its use of electronic platforms for data collection for a cross-sectional study, which might have led to selection bias and hindered direct cause-and-effect relationships, thereby limiting the results. Another limitation is that the self-reported measures used in this study may have increased the tendency for reporting bias. These limiting factors should be considered by future research to obtain more robust data and prevent such limitations. This project illuminates resilience as a psychological predictor of PPF. We did not collect data on other psychological factors, such as self-efficacy, self-esteem, and pain catastrophizing, which may also have affected the fatigue levels reported by mothers during postpartum. In addition, factors such as nutritional intake patterns and eating habits could affect the fatigue levels reported by mothers during this period. Further studies are needed to investigate the potential associations between these factors and PPF. Moreover, we did not consider the effects of a child’s medical condition on a mother’s fatigue level, as taking care of a child who requires hospital visits or needs careful attention at home may exacerbate the mother’s mental and physical fatigue. Future research should focus on the impact of the child’s health on the mother’s PPF. We also did not consider the effects of complications during labor, hospitalization due to postpartum complications, or even the period between the last and current pregnancy on a mother’s fatigue level. Such factors could contribute to PPF and affect a mother’s overall health. We thus encourage future research to thoroughly explore these matters. Finally, the paucity of evidence regarding the association between fatigue and resilience is a limitation of this study.

## 5 Conclusion

Given the deleterious effects of PPF on the health outcomes and general well-being of mothers during the stressful postpartum period, mothers should be regularly screened to identify factors associated with fatigue. Although our results show that BMI, sleep quality, depression symptoms, and resilience are predictors of PPF, our research provides empirical evidence that resilience is an important predictor of fatigue during the postpartum period. Such factors should be considered while building rehabilitation interventions for these mothers to improve their physical functioning and promote health. Fatigue affects both daily living and maternal activities. Focused rehabilitation interventions that target fatigue during this significant period also improve physical fitness and participation in activities and thus discourage non-communicable diseases such as cardiovascular problems. Thorough physical and mental examinations should be conducted to evolve the rehabilitation therapies recommended for mothers and therefore improve women’s health and physical and mental well-being during the significant postpartum period.

## Data availability statement

The raw data supporting the conclusions of this article will be made available by the authors, without undue reservation.

## Ethics statement

This study was conducted in accordance with the guidelines of the Declaration of Helsinki. The Ethics Committee of the Faculty of Medical Rehabilitation Sciences (FMRS), King Abdulaziz University, Jeddah, Saudi Arabia reviewed and approved the study procedure (FMRS-EC2022-006). The introductory section of the survey briefly described the rights of the participants and the study protocol. In addition, mothers who wished to participate were asked to agree to the informed consent sentences that were also provided in the introductory section. The principal investigator’s contact information was provided so respondents could reach out with any queries.

## Author contributions

BB: Conceptualization, Data curation, Formal analysis, Investigation, Methodology, Project administration, Resources, Supervision, Validation, Visualization, Writing – original draft, Writing – review & editing. MDA: Conceptualization, Data curation, Formal analysis, Investigation, Methodology, Resources, Validation, Visualization, Writing – original draft, Writing – review & editing. MIA: Conceptualization, Data curation, Formal analysis, Investigation, Methodology, Resources, Validation, Visualization, Writing – original draft, Writing – review & editing. FK: Conceptualization, Data curation, Formal analysis, Investigation, Methodology, Resources, Validation, Visualization, Writing – original draft, Writing – review & editing.
